# Mycotoxin Determination in Foods Using Advanced Sensors Based on Antibodies or Aptamers

**DOI:** 10.3390/toxins8080239

**Published:** 2016-08-12

**Authors:** Lin Xu, Zhaowei Zhang, Qi Zhang, Peiwu Li

**Affiliations:** 1 Oil Crops Research Institute of the Chinese Academy of Agricultural Sciences, Wuhan 430062, China; 15165385226@126.com; 2 Key Laboratory of Biology and Genetic Improvement of oil Crops, Ministry of Agriculture, Wuhan 430062, China; 3 Key Laboratory of Detection for Mycotoxins, Ministry of Agriculture, Wuhan 430062, China; 4 Laboratory of Risk Assessment for oilseeds Products (Wuhan), Ministry of Agriculture, Wuhan 430062, China; 5 Quality Inspection and Test Center for oilseeds Products, Ministry of Agriculture, Wuhan 430062, China

**Keywords:** mycotoxin, aptamer, antibody, sensor

## Abstract

Mycotoxin contamination threatens health and life of humans and animals throughout the food supply chains. Many of the mycotoxins have been proven to be carcinogens, teratogens and mutagens. The reliable and sensitive sensing methods are requested to monitor mycotoxin contamination. Advanced sensors based on antibodies or aptamers boast the advantages of high sensitivity and rapidity, and have been used in the mycotoxin sensing. These sensors are miniaturized, thereby lowering costs, and are applicable to high-throughput modes. In this work, the latest developments in sensing strategies for mycotoxin determination were critically discussed. Optical and electrochemical sensing modes were compared. The sensing methods for single mycotoxin or multiple mycotoxins in food samples were reviewed, along with the challenges and the future of antibody or aptamer-based sensors. This work might promote academic studies and industrial applications for mycotoxin sensing.

## 1. Introduction

Mycotoxins are secondary toxic metabolites primarily produced by fungi in food production [1]. These mycotoxins include, but are not limited to, aflatoxins (AFs), zearalenone (ZEA), deoxynivalenol (DON), ochratoxin (OTA) and T-2 toxin (a trichothecene mycotoxin). They are one major threat to the life and health of humans and live stocks [2]. Aflatoxin B_1_ (AFB_1_) is one highly toxic food contaminant and has been classified as a known human carcinogen (Group 1) [3]. ZEA and its metabolites can affect the estrogen secretion, especially during the mammalian reproductive process [4]. OTA is proved to be a nephrotoxic factor [5]. T-2 toxins can inhibit both the synthesis of protein and the synthesis of DNA and RNA [6]. There have been enormous medical costs caused by mycotoxin-based diseases, while mycotoxins are responsible for large economic losses in international trade [7]. Moreover, it is hard to reduce the risk of mycotoxins’ general prevention and control strategies because mycotoxins occur naturally and can contaminate the food throughout the food chain. Under certain temperature and moisture, mycotoxins can be found in food process, transport and storage [8].

In order to ensure the food safety against mycotoxins, sensitive and reliable determination strategies are requested [9]. There have been many efforts related to mycotoxin determination, based on lab-dependent methods and lab-independent methods [10,11,12]. For lab-dependent methods, typically, there are high performance liquid chromatography and gas chromatography, along with fluorescent detectors or mass spectrometry detectors [13,14]. These lab-dependent methods, as arbitrators, have merits of high sensitivity and stability, while having disadvantages of the high cost of equipment, labor and time. For lab-independent methods, they are more suitable for on-site monitoring of the mycotoxin risk in food. Among several lab-independent determination methods, antibody or aptamer based sensors have attracted more and more attention because of their advantages of high sensitivity and specificity, high throughput, portability and reusability [15,16,17]. For example, microfluidic devices, microarrays, and lateral flow strips can provide various sensing formats, depending on different applications. Among them, lateral flow strips could be the most successful device by using the naked eye or a reader [2]. For microarrays, it can allow the simultaneous determination of multiple targets that benefit its high throughput. Microfluidic chips could be a promising alternative due to their compact format that integrates series determination processes in one chip [18]. All of these sensing formats have been extensively studied in the past years.

There have been several critical reviews on the topic of sensors with respect to food safety [19,20,21]. However, few works focused on mycotoxin sensing. In this work, we focus on mycotoxin sensing based on antibodies or aptamers, with the aid of test strips, microarrays and microchips. The sensing strategies and their applications were discussed in detail, respectively. This work is supposed to provide updated information for the research and application of determination of mycotoxins based on antibody or aptamer sensors.

## 2. Sensing Strategies

### 2.1. Optical Sensing

#### 2.1.1. Fluorescence Sensors

Among diversified optical detection methods, fluorescence sensing methods are generally based on fluorescence quenching and recovery. Nanomaterials play an important role with respect to the enhancement of the sensitivity in the fluorescence recovery strategy. Colloidal gold nanoparticles [22], dendrimers [19], quantum dots [23], and graphene oxide [24] have been employed in the mycotoxin sensing [25]. In combination with nanoparticles, specific aptamers were also used to develop fluorescence sensors for mycotoxin detection [26,27]. Specifically, the complementary sequence of the aptamer was modified with a quencher and quenching was conducted by hybridization. Then, in the presence of the mycotoxin, the aptamer was captured by mycotoxins forming a G-quadruplex. Thus, the fluorophore-labeled aptamer was released from the quencher-modified complementary sequence to provide a fluorescence signal [26,28]. Other fluorescence aptasensors can be based on a displacement or competition assay [3,29,30]. The fluorophore-labeled aptamer is wrapped on the nanomaterials (e.g., single-walled carbon nanotubes, graphene oxide) and fluorescence would be quenched via energy transfer from the fluorescence tag to the nanomaterials. In the presence of the mycotoxin, the fluorophore-labeled aptamer switched from a random coil to an anti-parallel G-quadruplex, resulting in the fluorescence determination.

Fluorescent strip sensors have been developed for mycotoxin sensing [31]. They possess the advantages of easy operation, reduced determination time, low cost, portability and disposability [2,32]. They contain several typical parts, that is, base support, sample pad, conjugate pad, incubation-detection pad and absorbent pad [33]. By using capillary force, the mycotoxin can be sensed on the test line. Compared with the traditional nano-gold based test strip, fluorescent strip sensors allow higher sensitivity and reliability.

Some fluorescence sensing assays are based on fluorescence resonance energy transfer (FRET) [24]. In this manner, one chemical group serves as a donor to provide energy and the other one acts as an acceptor to accept the transferred energy. In order to achieve FRET, the excitation of the acceptor and the emission of the donor should have a big overlap. When the distance between donor and acceptor is close enough, the energy will transfer from the donor to the acceptor. More interestingly, the acceptor and donor in the FRET system can be designed in a biunique or one-to-multiple manner, providing the potential application of multiple mycotoxin sensing methods.

#### 2.1.2. Surface Plasmon Resonance Sensor

Surface plasmon resonance (SPR) allows the rapid and real-time analysis of a unique reusable sensing platform without any cleanup steps. As a label-free sensing technique, it is based on the changes in the refractive index of a material covering a metal surface [34,35]. SPR sensing can be used in the competitive inhibition assay and sandwich assay to determine mycotoxins. In competitive inhibition format, mycotoxin–protein conjugates (antigen) can be loaded on the activated SPR chip surface [36,37]. After the injection of sample-antibody mixture solution, the competition can be found between immobilized antigen and free mycotoxin in the sample. If mycotoxin is absent, the antibody combines with the immobilized antigen on the SPR chip surface. The concentration of mycotoxin in the extract solution is inversely proportional to the antibodies tethered to the sensor chip. In a similar fashion, other microchip formats have been widely used in sensing mycotoxins due to their advancements of fabrication [38]. It may be owed to the rapid development of emerging materials and easy fabrication of microchips, including glass, polymers and even paper [39,40]. In sandwich format, the antibody was added to the sample to recognize and react with the target to form the primary complex [34,41,42]. In the second step, the primary complex was injected to the sensor chip to generate the sandwich complex for the signal.

#### 2.1.3. Optical Waveguide Light Spectroscopy (OWLS) Sensor

As a label-free fluorescent sensing method, this method can precisely record the resonance angle of polarized laser light, after it is diffracted by a grating and incoupled into a thin waveguide [43]. OWLS can measure mycotoxin antigen adsorption [44] and have recently been employed to detect mycotoxins in milk samples [45]. The basic principle of OWLS is that linearly polarized light (He-Ne laser) is coupled into an optical waveguide via an optical grating and the incoupling only occurs at two well defined angles. These incoupling angles depend on the refractive index change within the evanescent field generated above the covered waveguide surface. By varying the incidence angle of the light, various mode spectra were obtained and these incoupling angles and incoupling light intensity are monitored for the determination.

Usually, an OWLS measurement system includes an optical grating, a readout instrument containing data collection software, a temperature controller and a flow injection analyzer. The OWLS could be used for various target determinations by immobilizing substrates specific to the target of interest on waveguide surface. High sensitivity and selectivity detection methods could be developed by combining immuno-recognition with specific and sensitive waveguide surface [46].

### 2.2. Electrochemical Sensing

#### 2.2.1. Amperometric Sensor

**Figure 1:**
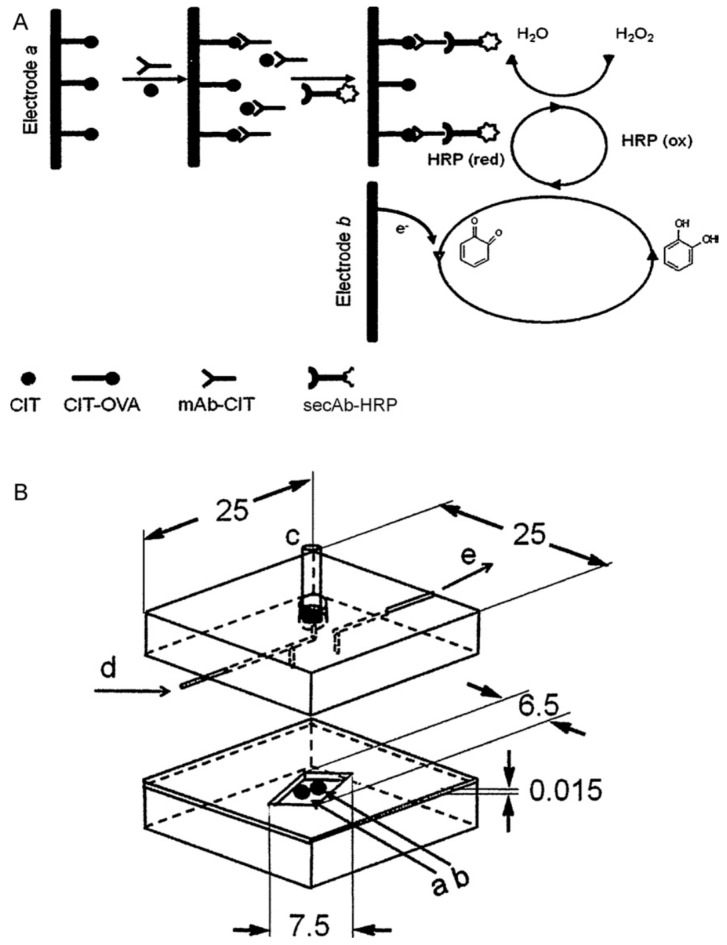
(**A**) Schematic representation of the citrinin immunosensor based on electrochemical detection using competitive assays; (**B**) schematic representation of the microfluidic immunosensor cell [18] (Reprinted from reference [50], Copyright (2011), with permission from Elsevier).

Amperometric immunosensors have become a widely-used technique for the detection of contaminants in food, mainly based on the ELISA technique [47]. With the advantages of rapid, sensitive and selective quantification, this method has been employed in the determination of mycotoxins in real agro-food matrices without complex sample preparation. For example, immunosensors based on chrono-amperometric measurement have been developed for OTA detection in this assay, reference and counter electrodes were designed on a polyester film, and working electrode was then activated for the further manufacture. After capture probe immobilization, the measurement was performed based on the relationship between anodic peak current of the oxidation product of OTA generated in enzymatic reaction [48,49]. Arevalo et al. constructed a microfluidic electrochemical immunosensor for citrinin (CIT) detection in rice samples [50]. This immunosensor contains two parts (as shown in Figure 1). The stainless-steel top body, as the counter electrode, included internal holes and channel for communication with reference electrode. Another part was the insulating bottom as the working electrodes. The current generated from the oxidation product was used for the quantification of the CIT in the rice sample with no sample pre-treatment.

#### 2.2.2. Potentiometric Sensor

**Figure 2:**
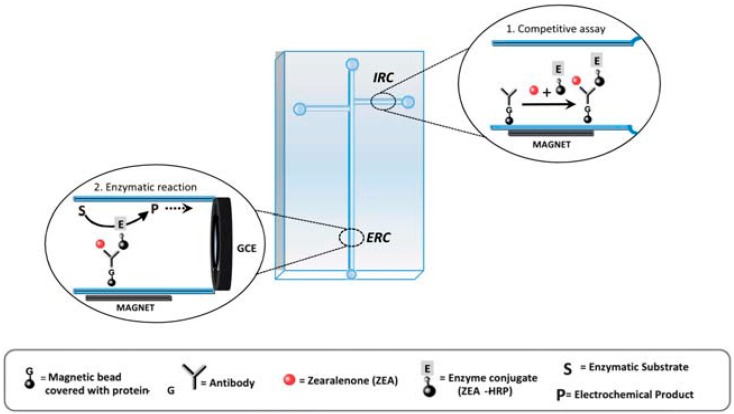
Microfluidic layout and immunoassay principle (IRC = immunological reaction chamber and ERC = enzymatic reaction chamber) [18] (Reprinted from reference [18], Copyright (2011), with permission from The Royal Society of Chemistry).

Both differential-pulse voltammetry (DPV) and cyclic voltammetry (CV) are employed in mycotoxin sensing. Differential-pulse voltammetry (DPV) is used to reduce the effect of the charging current in the sample measurement by detecting the current before potential change. DPV can serve to sense both single mycotoxins and multiple mycotoxins [51,52]. Another application used an electrochemical aptamer-sensor for OTA detection in real wheat starch samples achieving an LOD of 1.0 pg/mL [53]. DPV was generated with the signal amplification, which was achieved by the formation and simultaneous release of the aptamer-OTA conjugate with exonuclease digestion. Then, signal variance prior to and after the OTA addition, was detected for OTA quantification. Cyclic voltammetry (CV) method has also been used for mycotoxin measurements [54]. Integrating magnetic bead-based immunoassay on microfluidic chips, Hervas et al. made a double-T microchip for ZEA monitoring of infant foods [18]. An example is shown in Figure 2. In this CV based assay, there is a negative correlation between the signal and the mycotoxin concentration. Based on competitive binding between the ZEA and enzyme-labeled derivatives to a specific antibody, quantitative detection was achieved by the addition of electrochemical mediators and enzymatic substrates. Using both channels as immunologic and enzymatic reaction chambers, non-specific adsorption can be avoided.

#### 2.2.3. Impedimetric Sensors

The impedimetric immunosensors are used for mycotoxin sensing because of the capability of identifying and separating various contributions from the dielectric and electric response of the material [55]. By using an impedimetric sensing method, an immunoelectrode was fabricated via the co-immobilization of BSA and the antibody onto a gold substrate, followed by a self-assembled monolayer of 11-amino-1-undecanethiol (AUT) [55]. In the presence of mycotoxin, the value of transfer resistance was positively correlated with the increased concentration, allowing the sensitive detection for the OTA. Based upon the relationship between analyte concentration and charge transfer resistance (R_CT_) values obtained from electrochemical impedance spectroscopy (EIS), a high sensitivity impedimetric immunosensor was fabricated for OTA sensing in coffee samples [55]. The AUT modified gold surface was beneficial to the immobilization of antibody. Thus, it can improve the sensitivity by enhancing the electron transfer

## 3. Applications

### 3.1. Aflatoxin Sensing

Aflatoxin, a highly toxic metabolite of *Aspergillus flavus* and *Aspergillus parasiticus* species, has drawn greater attention due to its cytotoxicity and carcinogenicity. AFB_1_ is the most common aflatoxin, most likely to be found in cereal grains. AFM_1_ can be found in the milk of dairy cows fed a diet contaminated with AFB_1_.

Many reports focused on the electrochemical immunosensing of AFB_1_, including impedance [56] and impedimetric immunosensors [57]. Based on the modified electrodes, Lin et al. [58] reported an enzymatic hydrolysate-induced displacement reaction with multifunctional silica beads doped with HRP-thionine conjugate for AFB_1_ sensing in peanut samples. The principle was the competitive-type displacement reaction on the basis of the affinity difference between enzymatic hydrolysate (glucose) and its analogue (dextran) for concanavalin A (Con A) binding sites. After the immobilization, the multifunctional silica beads on a dextran-modified sensing interface, a competitive-type immunosensing, was conducted by using Au-functionalized with AFB_1_-BSA conjugate and invertase as a tag. This group reported another non-conventional competitive electrochemical immunosensing of AFB_1_ within 5 min via a competitive-type immunosensing method [59]. Based on a target-induced displacement reaction with antibody-functionalized mesoporous carbon nanoparticles, the mesoporous carbon electrode was modified with electroactive thionine molecules and then a polyclonal anti-AFB antibody, allowing good electrochemical responses for AFB_1_ at 0.003 ng/mL.

A test strip was successfully used in aflatoxin sensing by proper readers, and offered promise for rapid, sensitive, and cost-effective quantitative detection aflatoxins sensing [60]. Shim et al. [61] investigated an aptamer-based test strip for AFB_1_ sensing. A competitive sensing format was conducted with biotin-modified aptamer specific to AFB_1_ and streptavidin/anti-Cy5 antibody in corn sample. After a 30-min sensing, LOD was found to be 0.3 ng/g. AFB_1_ was detected in rice with test strips by using gold nanoflowers as a signal amplification probe and a portable optical strip reader [62]. A linear range of 0.5–25 pg/mL with a half maximal inhibitory concentration at 4.17 pg/mL that was 10 times lower than that (41.25 pg/mL) of the traditional gold nanospheres on test strips.

Field-effect based sensors have attracted considerable attention. Ah et al. [63] demonstrated an interesting sensor based on the Si field-effect transistor (FET) for AFB_1_ sensing. The signal was enhanced by Au nanoparticle charges under dry sensing conditions during an indirect competitive immunogold assay. Another recent example [64] was based on a graphene field effect capacitive immunosensor for AFB with a lower LOD of 0.1 fg/mL. It was proven that quantum capacitance of reduced graphene oxide and effective electrical double layer capacitance dramatically improved the sensitivity. Its sensitivity was greater than 1.5 times that of previous reports [65]. Park et al. [66] developed a CMOS compatible Si photosensitive immunosensor with competitive assay approach based on a CMOS compatible Si photodiode integrated circuit. The signal of open circuit voltage was transferred to a drain/source current of the FET by the connection of photodiode and FET gate.

Some typical solution-based optical sensing methods were introduced, including labeled (such as FRET, carbon-dots fluorescence, etc.) and label-free methods (such as electrochemical quartz crystal microbalance, SPR, etc.). Li et al. [67] illustrated a label-free FRET immunosensor for mycotoxin determination. The intrinsic fluorescence of tryptophan residues in AFB_1_ antibodies at 280 nm was quenched upon binding of specific AFB_1_ ligands. The Fab fragment was effective quenched by AFB_1_, while emission from intact anti-AFB_1_ was only partially quenched by this mycotoxin. Wang et al. [22] used an aptamer sensor based on fluorescent nitrogen-doped carbon dots on AuNPs for AFB_1_ sensing. Using electrostatic interactions, the prepared N,C-dots were assembled on aptamer/AuNPs. With the aid of a portable reader, time-resolved fluorescence test strips were used for sensing aflatoxins [68] and AFM_1_ [69] in food within 12 min, with LODs of 0.16 µg/kg, and a linear range of 0.48–30.0 µg/kg.

For AFM_1_ immunosensing, many works focused on the electrochemical method. A DNA-based immunosensor was developed by Banitaba and coworkers [70]. The gold electrodes were modified layer-by-layer with a thiol-modified single stranded DNA (ss-HSDNA) probe that specifically bound AFM_1_, a self-assembled monolayer of cysteamine and gold nanoparticles. With K_3_[Fe(CN)_6_]/K_4_[Fe(CN)_6_], it showed the linear range of 1–14 ng/mL with an LOD of 0.36 ng/mL. Another strategy to improve the sensitivity was to use DNA-aptamer recognition and electrochemical impedance spectroscopy detection, allowing a linear range of 20 to 1000 ng/kg with an LOD of 1.15 ng/L [71].

### 3.2. Zearalenone Sensing

ZEA is primarily the product of *Fusarium graminearum*, *Fusarium culmorum*, *Fusarium cerealis*, and *Fuarium equiseti* species growth on cereal crops, such as wheat, corn, barley and so on. ZEA is a known estrogen agonist and can cause reproductive abnormalities [72].

Photoluminescent semiconductor quantum dot (QD) has received much attention for ZEA sensing. Using CdSe/ZnS core/shell QD, a non-instrumental qualitative fluorescent-labeled immunosorbent assay (FLISA) was developed for ZEA sensing in raw wheat samples [73]. The introduction of QD significantly enhanced its sensitivity, resulting in LOD of 0.03 ng/mL. Compared to traditional CdSe/ZnS QD, quantum-dot submicrobead (QB) with carboxyl groups has approximately 2800 times brighter luminescence, suggesting a higher sensitivity. Based on the QBs, an immunochromatographic assay (ICA) for ZEA sensing in corn samples was reported. The QB-ICA-based assay obtained an LOD of 0.0625 ng/mL, which is 5.6 times higher than that of the previously reported AuNP-ICA assay. Recently, based on CdSe/ZnS QDs, molecular imprinted optosensing material (MIOM) was used for ZEA sensing in corn, rice and wheat flours [3]. In order to conduct a safer sensing method, cyclodo-decanyl-2,4-dihydroxybenzoate (CDHB) was used as an alternative to a ZEA template. The quantification sensing method was obtained by the inverse linear relation between the fluorescence intensity of MIOM and ZEA concentration. Despite a time-consuming sample pretreatment, MIOM was used for ZEA determination for the first time.

There are only a few studies on microfluidics for ZEA determination. A sensitive microfluidic immunosensor based on direct competitive immunoassay was constructed for rapid quantification of ZEA in feedstuff [74]. The analyst in the sample was capable to compete with ZEA-horseradish peroxidase (HPR) to bind to the anti-ZEA antibody. A double-T microchip layout electrochemical immunoassay was developed for the discrimination of ZEA in contaminated infant food samples [18]. The double-T layout served as chambers for the immunological and enzymatic reactions. By comparison with a calibration curve, the integrated sensor greatly simplified the operation procedure and reduced the reaction time. Combining a microfluidic chip with electro-kinetics in a magnetic bead-based electrochemical immunoassay, this “lab-on-a-chip” immunoassay was able to determine ZEA in both solid and liquid food samples.

Lei et al. demonstrated a label-free amperometric immunosensor based on mesoporous carbon (MC) and Au@AgPt nanorattles (Au-core and imperfect AgPt-shell structure) for ZEA ultrasensitive determination [75]. Au@AgPt nanorattles was immobilized on the MC and significantly enhanced the electron transfer capacity because of the synergistic effect of the Au, Ag and Pt nanoparticles. Anti-ZEA antibody was loaded onto Au@AgPt and cyclic voltammetry and square wave voltammetry were employed for ZEA measurement. The decreased peak current is proportional to ZEA concentration.

### 3.3. Ochratoxin A Sensing

OTA is produced by *Aspergillus ochraceus* and other srtains of *Aspergillus niger* and *Penicillium verrucosum* [55]. OTA is a Class 2B carcinogen and it has immunotoxicity and mutagenic effects [76,77].

Microfluidics, microarray and test strip techniques have been used for OTA immunosensing. Coupling with optical sensing stratergy, some immunosensors were reported. Based on indirect competitive immunoassay format, a regenerable glass immuno-biochip was developed using an automated microarray chip reader with chemiluminescence detection [78]. All assay steps of this immunosensor are automatized, which has potential for on-the-spot determination. The OTA conjugate was functionalized with a water-soluble peptide for covalent immobilization on a glass biochip by means of contact spotting. It allowed for at least 20 assay-regeneration cycles. Besides using chemiluminescence, an integrated microfluidic device based on indirect competitive immunoassay was developed for OTA quantification in wine samples. All the processes of extraction, concentration, detection and quantification can be performed in the integrated microfluidics [79]. Using competitive immunoassay format, an example based on aptamerstrip was developed for semi-quantitative OTA detection [80]. The sensitivity of the aptamer-based strip was better than that of conventional antibody-based strips within 10 min. The microfluidic aptamer immunosensor was further combined with an embedded SERS (surface-enhanced Raman spectroscopy) 2D platform for OTA sensing [81]. Specially, a microfluidic system for rapid and sensitive quantification determination of OTA in apple samples within 16 min was based on 3-aminopropyl-modified magnetic nanoparticles [82].

The energy transfer principle has attracted high interest. By using energy transfer, several reports reported were based on chemiluminescence resonance energy transfer (CRET) [83] or FRET [84]. For example, using the intrinsic fluorescence properties of anti-OTA antibody and OTA, a label-free, direct and noncompetitive homogeneous FRET immunosensor was developed [84]. In this system, the FRET signal generated by the OTA and anti-OTA binding was employed for the quantification and an LOD of 1 ng/mL was obtained. In order to improve the sensitivity, a FRET-based dual-emission ratiometric fluorescent aptasensor was based on a dual mode of fluorescent sensing (green-emitting CdTe QDs as donor) and onsite visual screening (red-emitting CdTe QDs) for the first time [85]. The green-QDs and gold nanoparticle (as accepter) were close enough with green-emitting CdTe QDs during the hybridization reaction, inducing FRET, and an LOD of 1.67 pg/mL was obtained.

SPR or localized SPR (LSPR) can be used for OTA sensing. An aptamer biosensor chip was developed based on SPR for OTA detection in wine and peanut oil [35]. The quantification was based on the straightforward direct binding assay. A regeneratable LSPR aptasensor for OTA sensing was based on a gold nanorod (GNR) and an OTA aptamer [86]. During OTA binding with aptamer, it was recorded by using a longitudinal wavelength shift of the LSPR peak in accordance with a change in the local refractive index near the GNR surface, due to the accumulation of OTA and G-quadruplex structures of OTA aptamers. The reuse of this LSPR aptasensor over seven times was achieved by heating in methanol at 70 °C to remove OTA.

The single-walled carbon nanotubes [29] (SWCN) and graphene oxide [30] were used for the turn-off sensing method. PVP-protected graphene oxide provided a lower detection limit by two orders of magnitude in comparison with bare graphene oxide [30]. On the other side, as a turn-on method, based on the basis of Tb^3+^, structure-switching aptamer and magnetic beads (MBs), a turn-on fluorescent aptasensor was developed for the label-free determination of OTA in wheat [87]. The OTA-aptamer G-quadruplex released two single-stranded signal probes. The single-stranded oligonucleotides greatly enhanced the emission of Tb^3+^ in solution, thus improving the sensitivity to 20 pg/mL. Magnetic nanoparticles were introduced as a purification process to enhance the sensitivity. A sensitive magnetic-fluorescent-targeting aptasensor was manufactured for one-step detection of OTA [88]. The magnetic-fluorescent-targeting was fabricated by the hybrid reaction between aptamer immobilized on the magnetic beads and the complementary sequence modified fluorescent nanoparticles. In the presence of OTA, fluorescent particles would be partially released due to the competitive binding between OTA and specific OTA aptamer. For wider linear range and higher sensitivity, Yao et al. [89] developed a biosensor for the OTA measurement integrating magnetic nanoparticles with rolling circular amplification (RCA). Magnetic nanoparticles were used to decrease the signal via efficient separation, and RCA was used for the signal amplification. In addition, the introduction of magnetic nanoparticles can not only enhance the detection sensitivity but also avoid the time-consuming washing steps. Interestingly, to avoid weak restoration to the original DNA conformation after repeated uses, a portable optical OTA aptasensor was reported based on a reversible ligand-grafted biosensing surface [90].

An aptamer-based competitive electrochemical sensor was described for OTA determination in wheat samples [51]. Magnetic beads were used for separation and immobilized on disposable screen-printed carbon electrodes (SPCEs). Then, the reaction between the enzymatic product and substrate was detected with differential-pulse voltammetry. An antibody-based photoelectrochemical OTA sensor was fabricated by assembly of CdSe QDs sensitized anatase TiO-functionalized electrode via the layer-by-layer method [91]. With ascorbic acid as an efficient electron donor, the photogenerated holes under visible-light irradiation were scavenged. The sensitivity was significantly enhanced due to the band alignment of CdSe and TiO2 in electrolytes.

### 3.4. Deoxynivalenolsensing

DON is a mycotoxin produced from *Fusarium graminearum* and *F. culmorum* [92]. The ingestion of DON is associated with acute gastroenteritis and vomiting effects.

Some reports focused on the electrochemical immunosensor for DON sensing [93]. Based on the use of immunomagnetic beads (IMBs) coupled with recombinant anti-DON Fab fragment, Romanazzo [94] developed an enzyme-linked-immunomagnetic-electrochemical (ELIME) method for DON detection in cereal and cereal-based food samples. The recombinant anti-DON Fab fragment was used as recognition element and the eight magnetized screen-printed electrodes were employed as electrochemical transducers. Magnetic beads were functionalized by the DON conjugates (conjugate DON with *N*,*N*_2_ disuccinimdyl carbonated and carbamylated human serum albumin). After the competitive reaction, bound Fab fragment was loaded onto the magnetized working electrode. Enzyme substrate was then added to sense DON. Despite the cross-reactivity of 3-Ac-DON, it met the sensing requests of the DON-contaminated food samples. Based on real-time electrochemical profiling (REP), an electrochemical immunosensor was fabricated coupled with microfluidics system for DON determination in wheat grain [95]. The integrated microfluidic system can allow multiplexed amperometric sensing. In the addition of substrate, the DON concentration was proportional to the current response with the horse radish peroxidase as a label. Compared to the conventional ELISA, this system is faster, has the potential to be automated, and is a more user-friendly testing method.

Test strips were successfully used for DON sensing [96]. They contain four-parameter models and the whole test procedure occurred within 10 min, providing an LOD of 0.3 mg/kg and detection range of 0–5 mg/kg in wheat samples. This immunosensor exhibits adequate reproducibility and repeatability, which has potential to be applied as an HACCP (hazards analysis and critical control points) tool in the cereal food industry. The optical sensors were well investigated, including SPR and OWLS sensors. Maragos illustrated an immunosensor based on a biolayer interferometry coupled with colloidal gold signal amplification [97].

### 3.5. Multiple Mycotoxin Sensing

Some fungi can produce multiple mycotoxins, and food can be contaminated by multiple fungal species at the same time. Thus, the co-contamination in food becomes a major concern in food safety, requiring simultaneous sensing methods [98].

Different optical detection technologies, containing fluorescence, chemiluminescence and SPR, have been developed for the multiple toxins determination. The chemiluminescent method was employed for the rapid, easy-to-use and high sensitive sensing multiple mycotoxins. A multiplex enzyme-catalyzed chemiluminescent sensor was developed for sensitive quantification of AFB_1_ and type-B-fumonisins in maize flour samples [99]. It integrated the indirect competitive test strip format with a lenseless “contact” imaging configuration, and a charge-coupled device (CCD) camera. This proposal demonstrated an LOD of 6 µg/kg and 1.5 µg/kg for type-B-fumonisin and AFB_1_ within 30 min, respectively. An automated flow-through immunosensor was manufactured with a dedicated chemiluminescence readout, for the measurement of AFs, OTA, FB_1_ and DON in cereal samples [100]. Using this immunosensor, all procedures containing extraction, dilution, measurement and surface regeneration could be completed within 19 min. The microarray was able to be reused at least 50 times. Hu and his coworkers [101] illustrated a high-throughput fluorescent immunosensor combing nonfouling polymer brush with competitive immunoassay for the detection of AFB_1_, OTA and ZEA. Due to the low nonspecific protein absorption of the brush and the uniform protein loading, LOD of AFB_1_, OTA and ZEA were 4, 4, and 3 pg/mL, respectively.

Nanoparticles were used as the fluorescent probe for the fluorescence sensing of multiple mycotoxins. Wu et al. [102] demonstrated a competitive fluorescence immunoassay using antibody-modified upconversion nanoparticles as multicolor signal probes for the simultaneous detection of AFB_1_ and OTA in maize samples. The use of antigen-functionalized magnetic nanoparticles (MNPs) greatly improved the sensitivity and selectivity. The sensor provided an LOD of 0.01 ng/mL for AFB_1_ and OTA, similar to results from a commercially available ELISA kit. Besides upconversion nanoparticles, quantum dots were widely used as fluorescent labels. Using quantum dots with different fluorescent spectrums, multiple targets can be simultaneously detected by scanning at different wavelengths. An immunochemical sensing technique based on fluorescent immunosorbent assay (FLISA) was developed for simultaneous screening of multiple mycotoxins (DON, ZEA, AFB_1_, T-2 and FB_1_) in cereal [23]. The cut-off values were 500, 100, 2 and 100 µg/kg for DON, ZEA, AFB_1_ and T-2 toxins, respectively. Fluorescence-labeled aptamer was also used for the multiple mycotoxin detection based on the fluorescence recovery. By utilizing an aptamer-photonic crystal encoded suspension array, Sun et al. [103] reported a simple assay for the measurement of OTA and FB_1_. Different aptamer probes were immobilized on the array, and various fluorescence labeled complementary sequences hybridized to the corresponding aptamer to generate double DNA sequences. With the addition of mycotoxins, the fluorescence-labeled complementary sequence would be released from the double DNA hybrid, leading to the regeneration of the fluoresence. The LOD and linear detection range were 0.25/0.16 pg/mL and 0.01–1/0.001–1 ng/mL for OTA and FB_1_, respectively.

Immunosensing based on SPR has been extensively used in mycotoxin determinations as well [104]. Meneely et al. [105] developed a simple, rapid, specific and sensitive screening assay based on SPR for the simultaneous detection of T-2 and HT-2 toxins in breakfast cereals, wheat and maize-based baby food. Only a simple extraction procedure was requested for sample preparation. LODs were reported as approximately 25 μg/g for baby food, breakfast cereal and wheat. Competitive inhibition immunoassay was conducted with SPR for rapid screening of multiple mycotoxins. Via the immobilization mycotoxin-labeled BSA on the sensor chip, Tomoyuki and coworkers [37] fabricated a SPR immunosensor with a simple fabrication within 600 s, including immobilization, activation and blocking. By using a continuous flow microspotter device, a microarray was fabricated and obtained for ZEA and DON sensing in maize and wheat samples [36]. Two syringe pumps were used in the microarray either to deliver the sample extraction or to flush/flow the analyte solution through the chip surface.

## 4. Conclusions

Either antibodies or aptamers can be employed in the mycotoxin determination in foods using advanced sensors. In comparison to antibodies, aptamers possess high sensitivity and specificity, chemical stability, easy synthesis and regeneration. These studies demonstrated the potential of mycotoxin determination with an ultra high sensitivity. An important issue for mycotoxin determination in food samples is the matrix effect. There will be a challenge to reduce the matrix effect by using a simplified sample preparation, especially in determination of multiple mycotoxins. A generally simple and effective sample process would improve its reliability. Various determination formats, including microarrays, test strips, and microfluidic chips were developed. Microfluidic and microarray devices have huge potential for the construction of low-cost and reusable devices. Despite the wide range of applications in multiple mycotoxin determination, microfluidic and microarray sensors might lag in their commercialization. The high throughput and multiple-use properties of microfluidic and microarray sensors pave the way to the fabrication of an integrated sensing system.
